# A novel machine learning-derived four-gene signature predicts STEMI and post-STEMI heart failure

**DOI:** 10.17305/bb.2023.9629

**Published:** 2024-04-01

**Authors:** Jialu Yao, Yujia Zhou, Zhichao Yao, Ye Meng, Wangjianfei Yu, Xinyu Yang, Dayong Zhou, Xiaoqin Yang, Yafeng Zhou

**Affiliations:** 1 Department of Cardiology, The First Affiliated Hospital of Soochow University, Suzhou, China; 2 Department of Cardiology, Dushu Lake Hospital Affiliated to Soochow University, Medical Center of Soochow University, Institute for Hypertension of Soochow University, Jiangsu Engineering Laboratory of Novel Functional Polymeric Materials of Soochow University, Suzhou, Jiangsu Province, China; 3 Center for Systems Biology, Department of Bioinformatics, School of Biology and Basic Medical Sciences, Soochow University, Suzhou, China; 4 Suzhou Medical College of Soochow University, Suzhou, Jiangsu Province, China; 5 Department of Vascular Surgery, Gusu School of Nanjing Medical University, Affiliated Suzhou Hospital of Nanjing Medical University, Suzhou Municipal Hospital (HQ), Suzhou, Jiangsu Province, China

**Keywords:** ST-elevation myocardial infarction (STEMI), heart failure (HF), monocyte, machine learning, prediction model

## Abstract

High mortality and morbidity rates associated with ST-elevation myocardial infarction (STEMI) and post-STEMI heart failure (HF) necessitate proper risk stratification for coronary artery disease (CAD). A prediction model that combines specificity and convenience is highly required. This study aimed to design a monocyte-based gene assay for predicting STEMI and post-STEMI HF. A total of 1956 monocyte expression profiles and corresponding clinical data were integrated from multiple sources. Meta-results were obtained through the weighted gene co-expression network analysis (WGCNA) and differential analysis to identify characteristic genes for STEMI. Machine learning models based on the decision tree (DT), support vector machine (SVM), and random forest (RF) algorithms were trained and validated. Five genes overlapped and were subjected to the model proposal. The discriminative performance of the DT model outperformed the other two methods. The established four-gene panel (human leukocyte antigen-J [*HLA-J*], complement factor properdin [*CFP*], Syntaxin-11 [*STX11*], and nuclear transcription factor Y subunit C [*NFYC*]) could discriminate STEMI and HF with an area under the curve (AUC) of 0.86 or above. In the gene set enrichment analysis (GSEA), several cardiac pathogenesis pathways and cardiovascular disorder signatures showed statistically significant, concordant differences between subjects with high and low expression levels of the four-gene panel, affirming the validity of the established model. In conclusion, we have developed and validated a model that offers the hope for accurately predicting the risk of STEMI and HF, leading to optimal risk stratification and personalized management of CAD, thereby improving individual outcomes.

## Introduction

Coronary artery disease (CAD) is the leading cause of death worldwide, with 85% of cardiovascular deaths attributed to acute myocardial infarction (MI) and stroke [1]. ST-elevation myocardial infarction (STEMI) is the most severe type of AMI that indicates an utterly occlusive coronary artery thrombus, leading to apoptosis and necrosis of cardiomyocytes, inflammation, and myocardial fibrosis [2]. It is well recognized as a determinant of morbidity and disability globally [3], currently accounting for 25%–40% of MI presentations [3–5]. Several large-scale observations in recent decades revealed reciprocal trends of a progressive increase in heart failure (HF) incidence that paralleled a decrease in mortality after MI events [6, 7]. Although percutaneous coronary intervention (PCI), the current standard treatment for STEMI, has improved short-term survival rates [8], severe complications associated with MI, such as HF, can still significantly worsen the prognosis. Among patients with an MI history, HF triples the total mortality risk and quadruples cardiovascular mortality [9, 10]. This underscores the importance of early STEMI and HF risk prediction, as well as the value of diligent patient monitoring.

Although concentrations of plasma brain natriuretic peptide (BNP)/N-terminal pro-B-type natriuretic peptide (NT-pro-BNP) are significantly elevated in STEMI patients and those admitted for adverse outcomes like HF and are used widely in clinical practice, they have limitations. They lack specificity and are likely to show similar elevations in congestive HF, pulmonary disease, atrial fibrillation, renal disease, and cancer therapy [11, 12]. Unexpectedly, a very low level was observed in a subset of patients hospitalized for HF [13]. Therefore, there is still an urgent requirement for novel objective biological indicators for the early prediction of STEMI occurrence and the development ofpost-STEMI HF.

Peripheral blood mononuclear cells (PBMCs), mainly monocytes, are recruited to the heart from the spleen and participate in inflammation [14, 15], where they function as crucial regulators of cardiac remodeling after MI [16]. According to this, it would be feasible to put forward new proposals for associating biological traits of PBMCs with STEMI risk and prognosis.

**Figure 1.**
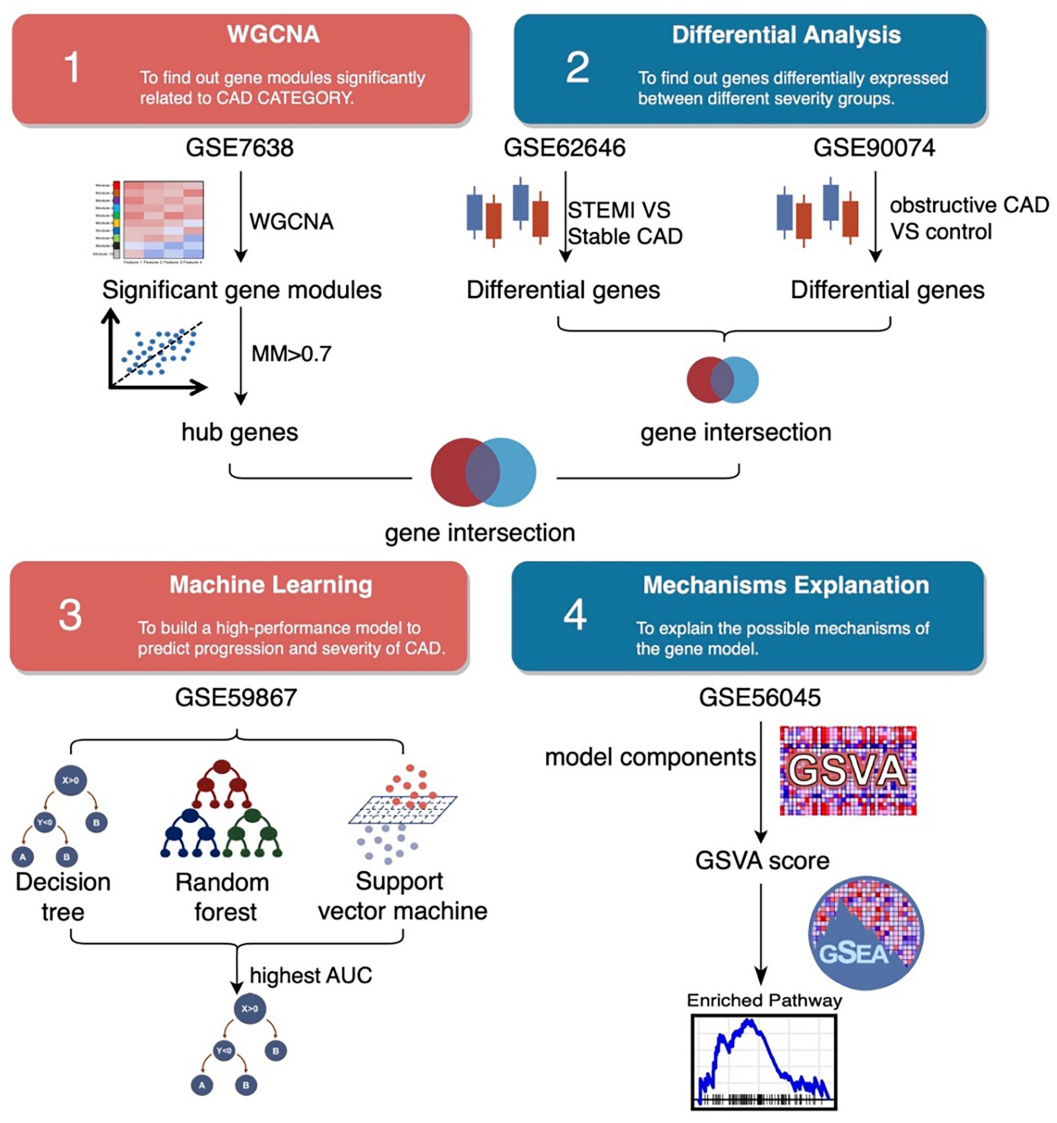
**Workflow of the study.** WGCNA: Weighted gene co-expression network analysis; STEMI: ST-elevation myocardial infarction; CAD: Coronary artery disease; GSVA: Gene set variation analysis; AUC: Area under the curve.

A rapidly growing body of omics data, comprised of many scattered studies by individual investigators, is worth mining to open up the possibility of linking specific biomarkers to corresponding pathological changes [17]. In this study, we hypothesized that key biomarker genes were identified through integrative molecular and clinical data across multiple monocyte cohorts and gene expression patterns. We sought to comprehensively evaluate extracted signature genes embodying STEMI risk. Here, we proposed a machine learning model based on these transcriptomic characteristics for early risk stratification of STEMI and post-STEMI HF. This novel blood-based gene panel may serve as an efficient prognostic tool for target-predictive, prevention, and personalized therapy for patients, cardiologists, and clinical investigators.

## Materials and methods

### Overall design

The workflow of this study is shown in Figure 1.

### Dataset collection and processing

Totally, five Gene Expression Omnibus (GEO) cohorts (GSE7638, GSE90074, GSE62646, GSE59867, and GSE56045) were included for bioinformatics analysis. The basic information for these datasets is summarized in Table 1 and Table S1. The GSE7638 dataset containing 160 samples with comprehensive clinical data was used for weighted gene co-expression network analysis (WGCNA). The GSE90074 and GSE62646 datasets from patients experiencing CAD and STEMI were used to screen differentially expressed genes (DEGs). Then, machine learning algorithms were performed to construct a prediction model based on the time-course expression data GSE59867. Finally, the large-scale transcriptional microarray data GSE56045 with 1202 samples was used for gene set enrichment analysis (GSEA). The “oligo” (Version 1.56.0) and “limma” (Version 3.48.3) packages were used in the workflow for processing Agilent, Illumina, and Affymetrix microarray [18, 19].

**Table 1:** Basic information of the dataset included in this study

**GSE ID**	**Sample size**	**Microarray platform**	**Usage**				
			**WGCNA**	**Differential analysis**	**Machine learning**	**GSVA**	**GSEA**
GSE7638	160	[HG-U133A_2] Affymetrix Human Genome U133A 2.0	*√*				
GSE90074	143	Agilent-014850 Whole Human Genome Microarray 4x44K G4112F		*√*			
GSE62646	98	[HuGene-1_0-st] Affymetrix Human Gene 1.0 ST		*√*			
GSE59867	353	[HuGene-1_0-st] Affymetrix Human Gene 1.0 ST			*√*		
GSE56045	1202	Illumina HumanHT-12 V4.0 expression beadchip				*√*	*√*

WGCNA: Weighted gene co-expression network analysis; GSVA: Gene set variation analysis; GSEA: Gene set enrichment analysis.

For the Agilent two-color microarray experiments (GSE90074), the raw data were first subjected to background correction and then “LOESS” within-array normalization, according to the suggested protocols for linear models to statistically assess differential expression. The resulting normalized log2 ratio (Cy5 Channel/Cy3 Channel) representing the test/reference was prepared for subsequent analysis.

For the Illumina single-channel expression beadchip data from the Multi-Ethnic Study of Atherosclerosis (MESA) project (GSE56045), the non-normalized matrix was subjected to “normexp” background correction, then quantile normalization, and finally, log2 transformed. The resulting matrix for the probe signal was prepared for subsequent analysis.

For the Affymetrix single-channel microarray data (GSE7638, GSE62646, and GSE59867), the CEL format raw files were annotated with the custom CDF files obtained from the Brainarray Microarray Lab (http://brainarray.mbni.med.umich.edu/Brainarray/Database/CustomCDF/, Version 24). The robust multichip average (RMA) normalization was performed to yield quantile-normalized signal intensity. The resulting log2 transformed value representing the expression level for each gene was prepared for subsequent analysis.

### Weighted gene co-expression network analysis (WGCNA)

WGCNA was performed based on the gene expression profiles of GSE7638 and corresponding clinical traits, including sex, age, collateral flow index, and CAD category (with or without CAD). Built-in functions of the “WGCNA” package (Version 1.70-3) were applied to fulfill complete exploratory data analysis and results visualization [20]. The WGCNA protocol can be summarized in the following steps. Initially, the expression matrix was sorted based on the median absolute deviation (MAD) value of each row (gene), and the top 5000 records were subjected to the subsequent WGCNA analysis. Then, briefly, a minimum power with *R*^2^ > 0.90 was set to optimize the power (β) for automatic network construction. The dynamic tree-cut method was implemented for module (cluster of densely interconnected genes) detection. The minimum module size (gene number) was set to 30. The association between the identified modules with clinical characteristics was assessed by Pearson’s correlation test. If the *P* value of the association was less than 0.01, the null hypothesis was rejected. Finally, the module membership (MM) score, indicating the likelihood of the membership of a gene in the corresponding module, was set at 0.70 to filter out potential false positives. The narrowed-down list of hub genes was subjected to further analysis.

### Identification of diferentially expressed genes (DEGs) and downstream analysis

Linear models and the empirical Bayes method were adopted for the normalized data matrix of GSE90074 and GSE62646 to assess differential expression statistically. If the *P* value was less than 0.01, the null hypothesis was rejected.

The web-based VennDiagram app (http://www.ehbio.com/test/venn/) was used for the overlap calculation for common genes. DAVID Analysis Wizard server (https://david.ncifcrf.gov/) was used to identify significantly enriched gene sets for DEGs [21]. If the *P* value was less than 0.05, the null hypothesis was rejected.

### Gene set variation analysis (GSVA) and functional enrichment

Gene set variation analysis (GSVA), a non-parametric and unsupervised method, was used for estimating the variation of gene set (gene signature candidates) enrichment through the samples of the GSE56045 dataset. The “GSVA” package (Version 1.42.0) was used to implement this operation [22]. Samples were stratified into two subgroups according to the mean GSVA score. The GSEA Desktop App (Version 4.2.2) was then used to computationally determine whether an a priori defined gene set (e.g., KEGG pathway, Gene Ontology Biological Process, and Chemical and Genetic Perturbations) shows statistically significant, concordant differences between the two subgroups (high GSVA score subgroup vs low GSVA score subgroup) [23]. If the *P* value was less than 0.05, the null hypothesis was rejected.

### Model construction with machine learning technologies

According to the split-sample validation approach, 353 MI and control samples of GSE59867 were randomly divided according to the 1:1 scheme into the model development and internal validation groups. The 307 PBMC samples of STEMI patients covered three time points (on the first day of MI, after four–six days of MI, and one month after MI). In addition, 72 HF progression and control samples of GSE59867 were used to test the models. Gene expressions depicted as attributes were input into machine learning methods, including support vector machine (SVM), decision tree (DT), and random forest (RF) algorithms to construct a model for STEMI and HF risk stratification. DT strategy was implemented with the “rpart” Package (Version 4.1.16). The SVM algorithm was fulfilled with the “e1071” Package (Version 1.7-9). RF method was carried out with the “randomForest” Package (Version 4.7-1) with the following parameters: ntree ═ 500 and mtry ═ 1. Receiver operating characteristic (ROC) curves were drawn to assess the prognostic value of the model. Area under the ROC curve (AUC) was calculated to assess the performance of the proposed model in separating positive and negative classes. An AUC below 0.75 indicates low discrimination accuracy, one ranging from 0.75 to 0.85 moderate accuracy, and one greater than 0.85 high accuracy.

### Statistical analysis

R language (Version 4.1.3) programs based on Bioconductor (Version 3.14) software packages were designed to perform all statistical and bioinformatics analyses.

## Results

### WGCNA identifies CAD risk factors

**Figure 2.**
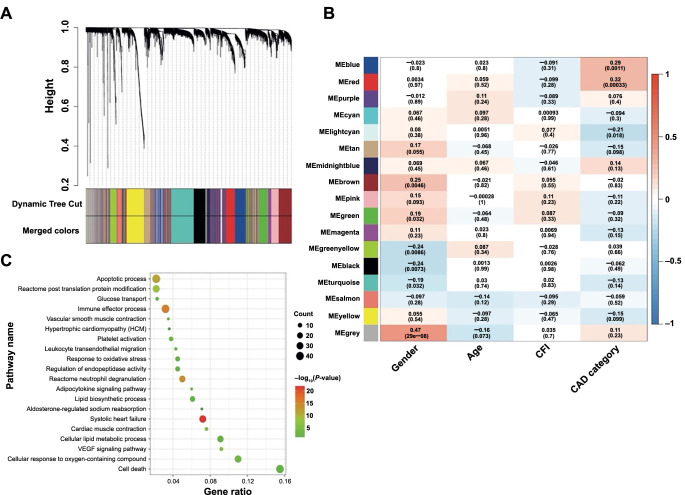
**WGCNA of GSE7638 dataset for CAD risk gene.** (A) Gene dendrogram determined by average linkage hierarchical clustering method. The color row underneath this dendrogram marks the module assignment identified by the dynamic tree-cut method. (B) Heatmap of Module-Trait relationships. A *P* value less than 0.01 rejects the null hypothesis. MEblue and MEred modules were significantly associated with CAD category (with or without CAD). (C) Functional annotation analysis for the 271 genes with MM > 0.70 from the two modules. MM: Module membership; WGCNA: Weighted correlation network analysis; CAD: Coronary artery disease.

The dataset GSE7638 was subjected to WGCNA to identify feature genes contributing to CAD risk (trait). Module-trait relationships were statistically analyzed upon completing the module assignment with the dynamic tree-cut algorithm (Figure 2A). The correlation coefficient and statistical significance of each correlation between coexpressed gene modules and clinical trait measurements were visualized in a heatmap. The depth of the shade color coded by the red–white–blue scale reflected the degree of positive (red) and negative (blue) correlation. As the statistics show, MEblue and MEred modules were positively associated with CAD risk (Figure 2B). Subsequently, 271 hub genes were further filtered with the MM cutoff of 0.7. Functional annotation analysis (Figure 2C) for these genes found significant enrichment for gene sets related to the cardiac disorder, such as apoptotic process, platelet activation, systolic HF, cardiac muscle contraction, response to oxidative stress, aldosterone-regulated sodium reabsorption. Additionally, metabolic processes, including the cellular lipid metabolic process, lipid biosynthetic process, glucose transport, and adipocytokine signaling pathway, were also significantly enriched.

### Differential analysis identified monocyte gene signatures for obstructive CAD and STEMI risk

**Figure 3.**
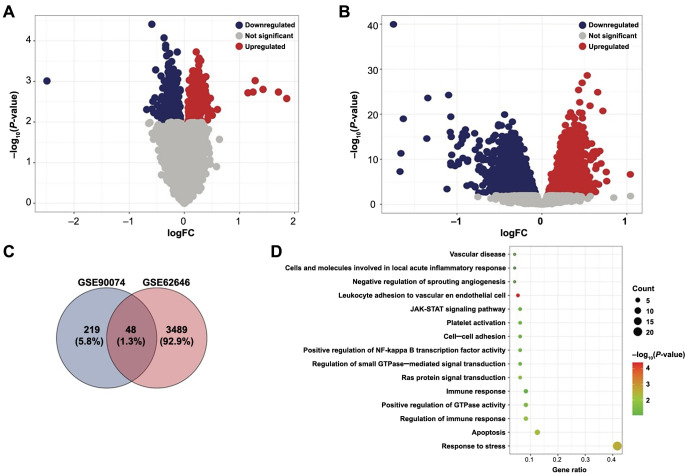
**Differential expression analysis identifying risk factors for obstructive CAD and STEMI.** (A) Volcano plot showing the significantly upregulated and downregulated genes of GSE90074 (obstructive CAD vs control); (B) Volcano plot showing the significantly upregulated and downregulated genes of GSE62646 (STEMI vs stable CAD); (C) Venn diagram showing the 48 genes upregulated in both datasets. No common downregulated gene was found; (D) Functional annotation analysis for the 48 genes. CAD: Coronary artery disease; STEMI: ST-elevation myocardial infarction.

To identify the risk or protective genes of CAD, we compared the mRNA expression of 93 obstructive CAD and 50 control samples retrieved from GSE90074. As shown, 267 genes were significantly upregulated, and 301 were downregulated (Figure 3A). Next, monocyte transcriptomics profiles of 14 stable CAD and 84 STEMI from GSE62646 were obtained and subjected to comparative analysis. Totally, 3537 upregulated genes and 3489 downregulated genes were identified (Figure 3B). Intersection shows that 48 genes were commonly upregulated in the GSE90074 and GSE62646 datasets (Figure 3C), but no commonly downregulated genes were found. Further functional annotation (Figure 3D) revealed that these myocardial disorder factors enriched cardiac pathogenesis pathways (e.g., JAK-STAT signaling pathway, Ras protein signal transduction, regulation of small GTPase mediated signal transduction, and positive regulation of GTPase activity) and relevant functional modules (e.g., apoptosis, platelet activation, cell–cell adhesion, vascular disease, cells, and molecules involved in local acute inflammatory response).

### Gene features predict myocardial infarct and heart failure risk

**Figure 4.**
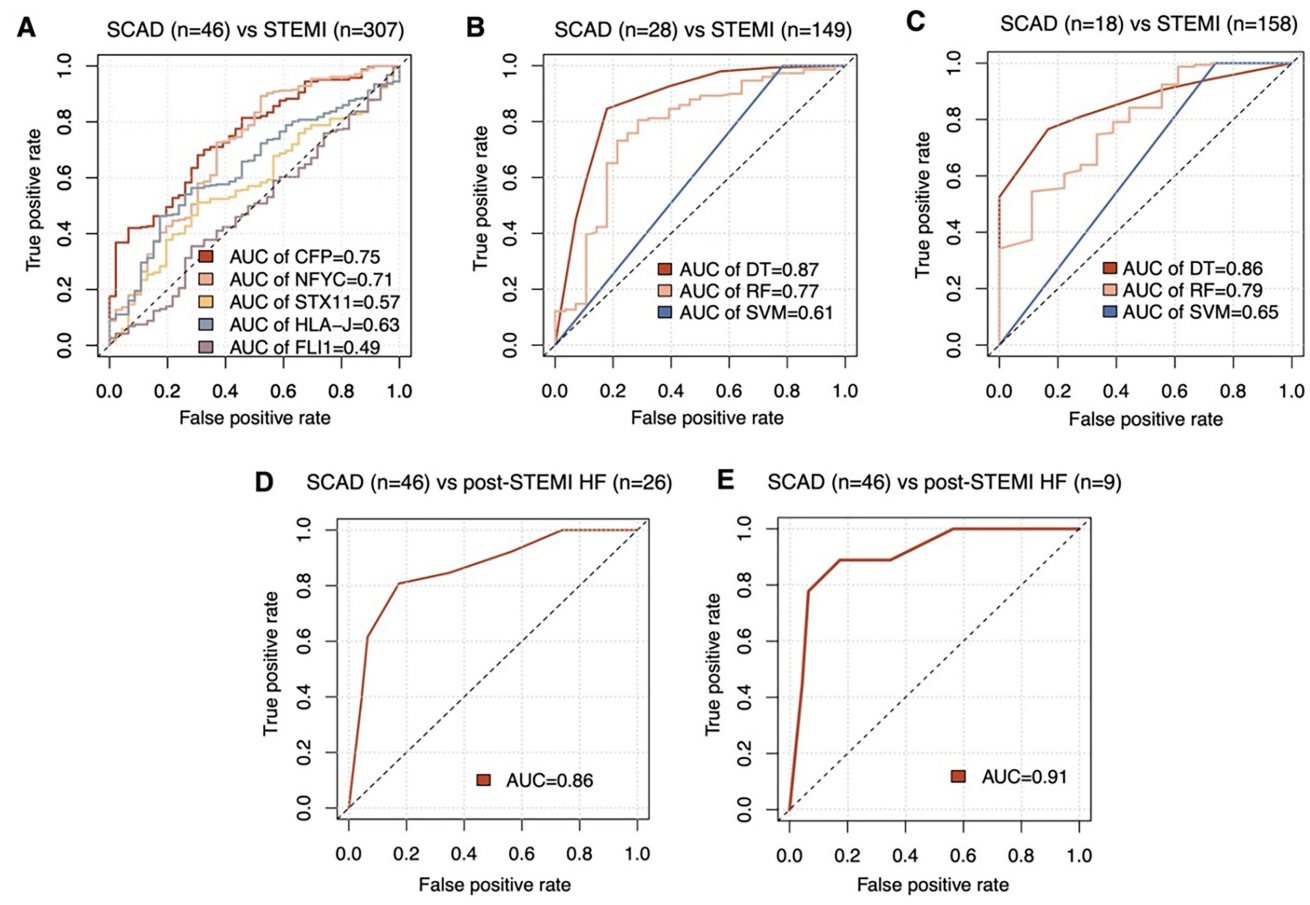
**ROC curve analysis assessing the prognostic value of different classifiers.** AUC was calculated to assess the performance. (A) Individual AUC for five genes (*HLA-J*, *CFP*, *STX11*, *FLI1*, and *NFYC*) were summarized in all 353 samples of GSE59867 dataset. GSE59867 samples were then randomly divided into training and validation subgroups. Performance evaluation of the models generated by DT, RF, and SVM was visualized for both training (B) and validation (C) subgroups. (D) Next, the four-gene model (*HLA-J*, *CFP*, *STX11*, and *NFYC*) proposed by the DT method was tested for its HF prediction in 72 samples (46 SCAD and 26 HF samples covered three timepoints: On the first day, after four–six days, and after one month of STEMI) of GSE59867. (E) Finally, this model was tested in the 55 samples (46 SCAD and 9 post-STEMI HF) obtained on admission of GSE59867. DT: Decision tree; SVM: Support vector machine; RF: Random forest; ROC: Receiver operating characteristic; AUC: Area under the ROC curve; HLA-J: Human leukocyte antigen-J; CFP: Complement factor properdin; STX11: Syntaxin-11; NFYC: Nuclear transcription factor Y subunit C; HF: Heart failure; STEMI: ST-elevation myocardial infarction; SCAD: Stable coronary artery disease.

**Figure S1.**
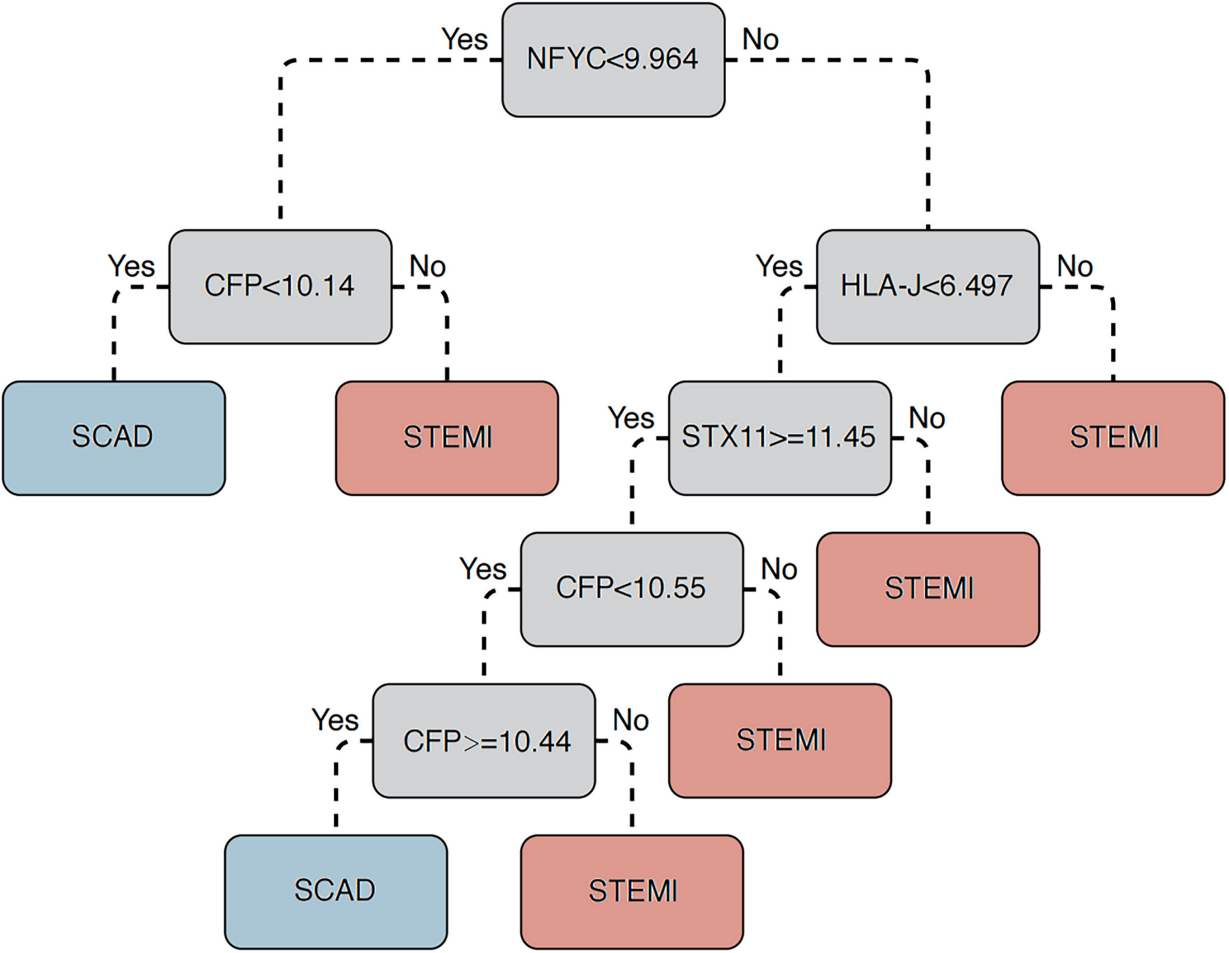
**Decision tree-based method for the integrating expression of the four genes to determine the risk of STEMI.** STEMI: ST-elevation myocardial infarction; HLA-J: Human leukocyte antigen-J; CFP: Complement factor properdin; STX11: Syntaxin-11; NFYC: Nuclear transcription factor Y subunit C; SCAD: Stable coronary artery disease.

To further narrow down the candidate list, the 271 CAD-associated genes identified by WGCNA were intersected with 48 genes commonly upregulated in GSE90074 and GSE62646. Totally, five genes, including human leukocyte antigen-J (*HLA-J*), complement factor properdin *(CFP*), Syntaxin-11 (*STX11*), Fli-1 proto-oncogene (*FLI1*), and nuclear transcription factor Y subunit C (*NFYC*), were contained within the intersection outcome (Table 2) and subjected to the model proposal. ROC plot analysis was used to assess the accuracy of classifiers for MI risk in the GSE59867 dataset (353 samples). However, none of the five genes (Figure 4A) could achieve high classification accuracy (as a rule of thumb, a ROC-AUC above 0.85). Then, three popular machine-learning strategies were adopted. To do so, the original dataset of GSE59867 was subgrouped into learning and validation sets with a 1:1 size ratio in a standard split-sample validation scheme. As an obvious result, only the classification by DT technique could yield an ROC-AUC greater than 0.85 in both learning (Figure 4B) and validation (Figure 4C) sets. Totally, four genes (*HLA-J, CFP, STX11*, and *NFYC*) constituted this DT prediction model (Figure S1). In the test for HF risk in GSE59867, 26 samples of HF collected at three time points (admission, discharge, and after 1 month) vs 46 stable CAD were used. This DT-based classifier attained an ROC-AUC of 0.86 (Figure 4D). Then, to further evaluate its performance as present-on-admission prediction, this DT model was tested in a cohort consisting of nine HF samples obtained on admission for STEMI and 46 stable CAD. In this case, the ROC-AUC value was 0.91 (Figure 4E). These results suggested that this DT model might have substantial potential for predicting STEMI risk and HF risk after STEMI.

### Gene set enrichment analysis (GSEA)

**Figure 5.**
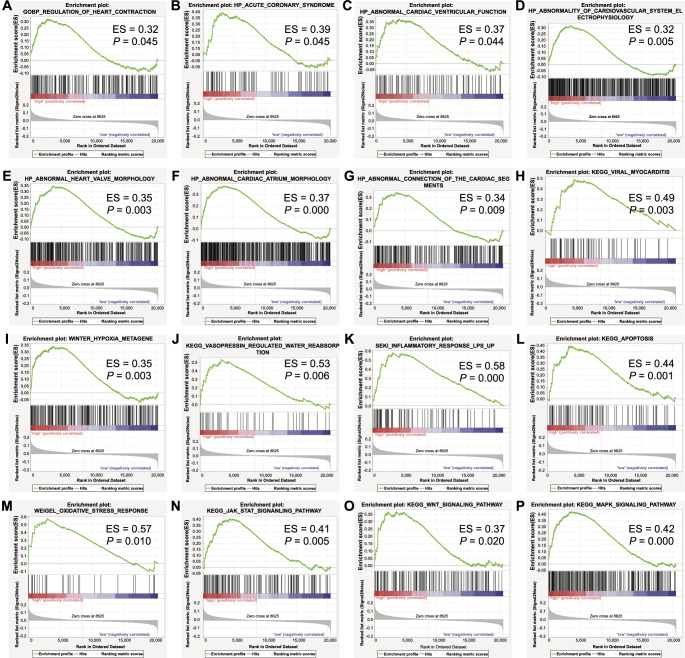
**GSEA plots showing the activation status of biological pathways in distinct gene signature patterns.** GSVA was performed to assess the variation of the gene expression (*HLA-J*, *CFP*, *STX11*, and *NFYC*) through the samples of the GSE56045 dataset. Samples were subgrouped according to the mean value of the GSVA score. GSEA was performed to computationally determine whether a gene set shows the statistically significant difference between subgroups with high and low GSVA scores. (A) GOBP_REGULATION_OF_HEART_CONTRACTION, (B) HP_ACUTE_CORONARY_SYNDROME, (C) HP_ABNORMAL_CARDIAC_VENTRICULAR_FUNCTION, (D) HP_ABNORMALITY_OF_CARDIOVASCULAR_SYSTEM_ELECTROPHYSIOLOGY, (E) HP_ABNORMAL_HEART_VALVE_MORPHOLOGY, (F) HP_ABNORMAL_CARDIAC_ATRIUM_MORPHOLOGY, (G) HP_ABNORMAL_CONNECTION_OF_THE_CARDIAC_SEGMENTS, (H) KEGG_VIRAL_MYOCARDITIS, (I) WINTER_HYPOXIA_METAGENE, (J) KEGG_VASOPRESSIN_REGULATED_WATER_REABSORPTION, (K) SEKI_INFLAMMATORY_RESPONSE_LPS_UP, (L) KEGG_APOPTOSIS, (M) WEIGEL_OXIDATIVE_STRESS_RESPONSE, (N) KEGG_JAK_STAT_SIGNALING_PATHWAY, (O) KEGG_WNT_SIGNALING_PATHWAY, and (P) KEGG_MAPK_SIGNALING_PATHWAY were significantly enriched. ES: enrichment score. A *P* value less than 0.05 is statistically significant. GSEA: Gene set enrichment analysis; GSVA: Gene set variation analysis; HLA-J: Human leukocyte antigen-J; CFP: Complement factor properdin; STX11: Syntaxin-11; NFYC: Nuclear transcription factor Y subunit C.

To uncover the underlying biological themes that are different between subjects with high and low expression levels of the four genes involved in the DT model, we performed pathway-level comparative analysis. The 1202 monocyte samples from the GSE56045 dataset were stratified according to the mean GSVA score of the four genes. Further, GSEA enrichment results showed significant enrichment of gene modules related to cardiac disorders, including heart contraction (Figure 5A), acute coronary syndrome (Figure 5B), abnormal cardiac ventricular function (Figure 5C), abnormality of cardiovascular system electrophysiology (Figure 5D), heart valve morphology (Figure 5E), cardiac atrium morphology (Figure 5F), connection of the cardiac segments (Figure 5G), and viral myocarditis (Figure 5H). In addition, biological processes involved in the pathogenesis of MI and HF, including hypoxia (Figure 5I), vasopressin-regulated water reabsorption (Figure 5J), inflammation response (Figure 5K), apoptosis (Figure 5L), and oxidative stress (Figure 5M) were also enriched. Moreover, signaling pathways instrumental in myocardial damage or cardioprotection, such as JAK-STAT (Figure 5N), Wnt (Figure 5O), and MAPK (Figure 5P), were overrepresented.

## Discussion

Diagnostic strategies should be established to prevent or detect MI and HF early in high-risk populations. Clinical prediction, based on mathematical models, is developed to estimate the probability that a particular disorder is present or to assess the likelihood that a specific adverse outcome will occur in the future [24]. Many efforts have been devoted to developing the biomarker model that stratifies CAD subjects according to their MI and HF risk. With the steady development of high-throughput technologies (e.g., microarrays, next-generation sequencing, and mass spectrometry), along with the accelerated accumulation of omics data and the substantial improvement in statistics and artificial intelligence (e.g., machine learning), several signatures based on transcriptomics, epigenomics, proteomics, and metabolomics have been identified assessing MI risk and prognosis [25–30]. However, some studies were plagued by small sample sizes, low classification accuracy (ROC-AUC less than 0.85), lack of validation, or even a failure to provide sufficient predictive information (e.g., statistics from ROC analysis). A recent study proposed the diagnostic value of a combination of five genes identified by logistic LASSO regression to discriminate STEMI patients from controls and nominated two genes as predictive biomarkers of post-STEMI HF. However, the robustness of this conclusion could be challenged by the heterogeneity of the sample source [25]. Another three-gene model for recognizing post-acute MI HF [30] suffered from data reuse and lack of validation, significantly impacting the generalizability. In this study, the cell-type homogeneity of the included datasets ensured the representativeness of our four-gene model. Furthermore, real-world representation was modeled through access to a diverse range of data sources. Moreover, our model’s performance was tested in both derivation and validation groups.

**Table 2:** Basic information of the five genes subjected to further characterization

**Symbol**	**Gene name**	**Entrez ID**
*HLA-J*	Major Histocompatibility Complex, Class I, J (Pseudogene)	3137
*CFP*	Complement factor properdin	5199
*STX11*	Syntaxin 11	8676
*FLI1*	Fli-1 proto-oncogene, ETS transcription factor	2313
*NFYC*	Nuclear transcription factor Y subunit gamma	4802

All genes listed in this table showed positive association with CAD risk (GSE7638), significant upregulation in obstructive CAD (GSE90074), and significant upregulation in STEMI (GSE62646). HLA-J: Human leukocyte antigen-J; CFP: Complement factor properdin; STX11: Syntaxin-11; NFYC: Nuclear transcription factor Y subunit C; STEMI: ST-elevation myocardial infarction; CAD: Coronary artery disease.

This study presents a strategy for a high-risk monocyte signature of adverse events after coronary heart disease derived from co-expression network analysis and differential analysis of transcriptomics data. Further, DT analysis proposed a multivariable prediction model for MI and HF, achieving good classification accuracy (ROC-AUC ranged from 0.86 to 0.91). This could refine risk prediction for adverse clinical outcomes beyond the current state-of-the-art and hopefully discriminate subjects with poor prognoses from those with stable coronary heart disease. While on the subject, it is worth noting that due to the limitation in gene coverage of the microarray platform, the prediction model should be further optimized when higher-throughput data are acquired. In addition, collateral flow index is a diagnostic measure used to evaluate the sufficiency of collateral circulation within the coronary arteries, serving as a key indicator of the CAD severity and playing a pivotal role in guiding therapeutic decisions for CAD, especially when revascularization procedures are under consideration [31, 32]. In this study, we did not identify any modules correlated with collateral flow index based on WGCNA, which we consider a potential subject for future research. Meanwhile, further data accumulation is bound to expand the application scope of this model to non-ST-elevation MI (NSTEMI).

The machine learning model built on *HLA-J, CFP, STX11,* and *NFYC* genes demonstrated great value for MI prediction. Besides, these four genes might be prognostic candidates for HF. *HLA-J* is a member of the human leukocyte antigen (HLA) gene family, pivotal in the immune system for its role in encoding cell surface proteins critical for immune responses. Recent studies have highlighted its prognostic significance in various conditions, including multiple sclerosis, breast cancer, and uveal melanoma [33–35]. *CFP* plays a role in regulating the complement system, essential in immune defense. It has been identified as a potential tumor suppressor in several malignancies, including breast cancer, lung cancer, and gastric cancer [36, 37]. *STX11* participates in crucial intracellular processes, such as vesicle trafficking and membrane fusion. Its deficiency is associated with familial hemophagocytic lymphohistiocytosis type 4 [38]. Furthermore, *STX11* has emerged as a novel tumor suppressor gene implicated in peripheral T-cell lymphoma [39]. *NFYC* is a transcription factor that regulates the epigenome and has been identified as an oncogene in choroid plexus carcinoma [40]. However, to our knowledge, the association between this four-gene signature and MI and HF has rarely been reported. Therefore, we performed an enrichment analysis for the biological pathway and functional gene set to explore its clinical and biological indications for the risk of MI and HF in patients with coronary heart disease. Not surprisingly, functional gene sets related to heart contraction, abnormal cardiac morphology, HF, myocarditis, hypoxia, vasopressin-regulated water reabsorption, and the inflammatory response were significantly overrepresented. Apoptosis contributes to cardiomyocyte cell death in MI and participates in the subsequent development of symptomatic HF [41]. Oxidative stress induced during MI leads to platelet reactivity and activation in the circulation, playing a role in atherothrombotic plaque formation, MI, and subsequent expansion [42]. Statistically significant enrichment of the above pathways drafted a big picture of the cascade of events leading to a high risk of MI and HF. Moreover, signaling pathways known to contribute significantly or be triggered during the pathogenesis of MI, including JAK-STAT [43], Wnt [44], and MAPK [45], were enriched. This preliminarily outlines the underlying molecular mechanisms in the evolution of this myocardial disorder. In short, the concentration of this enrichment is very consistent with what we observed in the modeling and validation studies, underlining the validity of the four-gene signature.

This study’s model construction has several merits. First, examining a small number of easily collected, low-cost peripheral monocyte predictors is naturally more maneuverable than tissue assays. Additionally, this study integrated multiple transcriptomics datasets from public databases, totaling 1956 individuals. We also avoided using the same dataset throughout the workflow repeatedly. The selected features (gene signature) input into model development were obtained from the intersection outcomes of multiple datasets, discovered by different technologies. The diversity of variable screening methods and data sources ensured the feasibility of the input features. This will support the optimization of STEMI prediction, prevention, and personalized medical strategy to improve individual outcomes.

Despite these advantages, this study has several limitations that need to be addressed in future studies. First, although this study has a split-sample validation (50% for model development and 50% for validation), it could still be considered as an internal validation. Ideally, a fully independent external validation is needed for further confirmation of its reliability. Moreover, the sample size for both model development and validation should be increased to optimize predictive capacity and achieve more satisfactory outcomes. Besides, the subject number for HF risk prediction was even smaller. Furthermore, all datasets included in this study originated from research conducted in Europe and the United States, which could inevitably lead to the uniform ethnicity composition of subjects (mainly Caucasian). Socioeconomic status, lifestyle, health care access, and genetic background may interact with the genes of interest and consequently impact specific phenotypes (e.g., disease risk and severity), thus influencing the generalizability of our conclusion. Last but not least, the stability of prediction performance could be impacted if the models were constructed and validated based solely on retrospective datasets [46]. In this study, all transcriptomics datasets used in gene signature discovery, model construction, and validation were retrieved from retrospective studies. For this reason, further multi-ethnic and large-scale prospective validations with strict clinical evidence-based data are warranted.

## Conclusion

Our results suggest that this four-gene signature has a predictive role in identifying CAD patients with an increased risk of STEMI and post-STEMI HF. This monocyte gene expression approach coupled with machine learning represents a promising strategy for enhancing risk stratification in CAD progression. Future research should further examine this approach based on monocyte biomarkers for risk stratification of NSTEMI.

## Supplemental data

**Table S1.** Details for the samples from public datasets

Available at the following link: https://docs.google.com/spreadsheets/d/1bELTbxrFqxqhqet640R2ikOXlsoIa01gwv8NoTXaJpA/edit?usp=sharing

## Data Availability

Public transcriptome datasets and corresponding clinical annotations were retrieved from NCBI GEO repository (https://www.ncbi.nlm.nih.gov/geo/).
